# Information Literacy in Food and Activity Tracking Among Parkrunners, People With Type 2 Diabetes, and People With Irritable Bowel Syndrome: Exploratory Study

**DOI:** 10.2196/13652

**Published:** 2019-08-01

**Authors:** Pamela McKinney, Andrew Martin Cox, Laura Sbaffi

**Affiliations:** 1 Information School University of Sheffield Sheffield United Kingdom

**Keywords:** activity logging, food logging, information literacy, irritable bowel syndrome, personal informatics, quantified self, running, self-tracking, type 2 diabetes

## Abstract

**Background:**

The tracking, or logging, of food intake and physical activity is increasing among people, and as a result there is increasing evidence of a link to improvement in health and well-being. Crucial to the effective and safe use of logging is a user’s information literacy.

**Objective:**

The aim of this study was to analyze food and activity tracking from an information literacy perspective.

**Methods:**

An online survey was distributed to three communities via parkrun, diabetes.co.uk and the Irritable Bowel Syndrome Network.

**Results:**

The data showed that there were clear differences in the logging practices of the members of the three different communities, as well as differences in motivations for tracking and the extent of sharing of said tracked data. Respondents showed a good understanding of the importance of information accuracy and were confident in their ability to understand tracked data, however, there were differences in the extent to which food and activity data were shared and also a lack of understanding of the potential reuse and sharing of data by third parties.

**Conclusions:**

Information literacy in this context involves developing awareness of the issues of accurate information recording, and how tracked information can be applied to support specific health goals. Developing awareness of how and when to share data, as well as of data ownership and privacy, are also important aspects of information literacy.

## Introduction

### Background

Self-tracking has been defined as *practices in which people knowingly and purposively collect information about themselves, which they then review and consider applying to the conduct of their lives* [1].

While manual recording of personal data has been advocated for over many years, the potential of digital devices and apps to monitor and measure your own data and then share that information with others has a huge potential for improving personal health [2]. Mobile phones are ubiquitous, powerful, and connected devices that are highly valued by users, and they have the potential to support healthy behaviors through their built-in sensors and downloadable apps [3,4]. Smartphone penetration is high, with 83% of people in the United Kingdom owning one [5] and 25% of smartphone app users regularly using a health or fitness app [6]. The act of tracking has been shown to be beneficial in terms of increasing desired behaviors in the health arena [7,8], and research has focused on the value of apps and technologies to support health goals in a variety of contexts (eg, menstrual tracking [9], management of migraines [10], diet and exercise [11,12] and chronic disease [13,14]).

Lifestyle changes supported by self-management for people with noncommunicable diseases are a key factor in their prevention and treatment [15]. It is accepted that to achieve health goals involving weight loss, people must address both diet (in terms of reducing calorific intake) and also increase physical exercise [16]. People who track both diet and physical activity are more likely to lose weight [11,17]. Wearable devices that automatically track physical activity, such as FitBit, are increasingly popular, with the market research organization Mintel estimating that 38% of UK consumers have an interest in wearable technology to monitor health and fitness [18].

However, there are a number of barriers to the effective and safe use of tracking, such as whether tracked data is accurate enough to be used by health professionals, ease of use of apps, and the information and digital literacies required to use them effectively. In addition, there is the threat to personal privacy from reuse of tracked data shared to third parties, and the problem that consumers still need to develop an understanding of the social norms of tracking data and of sharing said data [3,13,19]. Of particular interest to this paper is the way that levels of information literacy might be one important determinant of effective and safe use of tracking. Information literacy can be defined as *the ability to think critically and make balanced judgements about any information we find and use. It empowers us as citizens to develop informed views and to engage fully with society* [20].

Initially, research in information literacy focused on the educational context, but it has increasingly broadened towards developing an understanding of information literacy across a range of contexts, such as everyday life [21], the workplace [22] and health [23]. Information literacy is highly contextual, with the necessary set of skills, abilities and practices varying enormously depending on the setting [24]. Previous research into the information literacy aspects of diet and fitness tracking has shown a need for skills in a number of inter-related areas [25], such as: (1) understanding the importance of quality in data inputs; (2) interpreting tracking information outputs in the context of the limitations of the technology; (3) being aware of data privacy and ownership; and also (4) managing appropriate information sharing.

In order to investigate the role of information literacy in the safe and effective use of tracking in a range of contexts, we selected three contrasting populations to study: participants in parkrun free running events, people with type 2 diabetes, and people with irritable bowel syndrome (IBS). The populations were identified during a previous study as being inclined to want to support their health through tracking [25], and were selected to capture variations of underlying motivation, the need for tracking, and in tracking behaviors. An investigation across the three groups offers insights into the diversity of tracking practices.

### Information Literacy and Health

There is increasing interest in the contextual nature of information literacy, which, in the health field, is often referred to as health information literacy [26]. The interest in the relationship between information and health is driven by the increasing demand for such information among the population, as well as the changing nature of the relationship between people and healthcare providers [27]. Self-tracking could also be understood as a response to a growing perception of individual responsibility for health [28].

Phenomenographic studies have demonstrated substantial variation in conceptions of health information literacy, which can mean: (1) striving for or reaffirming wellness; (2) knowing or protecting oneself; (3) screening, storing, or creating knowledge; (4) using information to choose a treatment path; (5) paying attention to the body; or (6) participating in learning communities [26,27]. This variation underlines the complexity of both the concept of health information literacy, as well as the multiple and distinctive ways in which people engage with and use health information in their lives. Health literacy has been defined specifically within an electronic context as electronic health (eHealth) literacy and is understood as a transactional model which focuses on people’s ability to interact with technology, other users, and to apply information for improved health [29]. Understanding how people engage with and use health information, as well as develop their information literacy, is of interest to public health bodies as they attempt to design health messages that will have an impact on people’s behaviors [30]. The view that health information literacy is an example of a contextual application of information literacy is adopted in this paper [24].

### Food and Activity Tracking

Research has shown that use of apps can motivate people to adopt healthy behaviors, including a healthy diet, increased physical activity and weight loss [11,31]. Self-management of diet is seen to be a critical issue in some chronic disease management [32], and it has been found that mobile apps for dietary assessment are as valid and reliable as more traditional methods of food tracking [33]. The MyFitnessPal app, popular with both health professionals and the general public, has been found to promote positive changes to the lifestyles of people suffering from diabetes [16]. Tracking can give people a sense that they are taking control of aspects of their life, that they are developing enhanced self-knowledge and self-management, and that they have improved understanding of their own bodies [1,34]. Research has revealed different styles of personal tracking: (1) directive or goal driven tracking; (2) documentary tracking to simply record bodily information; (3) diagnostic tracking to link different aspects of behavior; (4) collecting rewards as a way to register achievement; and (5) fetishized tracking characterized by an interest in gadgets and technology [35]. People gain enjoyment from setting and achieving personal goals from tracked data [1], with use of multiple devices common among those who actively engage in tracking behavior [36].

However, there are a number of potential issues associated with tracking practices, including: (1) tracking can radically alter eating practices; (2) can cause a loss of pleasure in food [1]; (3) can remind people of the negative aspects of a chronic disease [13]; (4) users may fetishize data and develop unhealthy obsessions [1,12,13]; (5) apps tend to not to be based on any behavior change theory [37,38]; and (6) people can find the apps very time-consuming to use, leading to a culture of temporary use, which is particularly true if apps do not meet expectations [3,32,39]. In addition to these issues, there are a number revolving specifically around the information literacies required to make effective and safe use of tracking. Accuracy of data input in tracking is important, but people recognize that their own recording practices may not be sufficiently diligent [39]. People who use apps should have concerns around their ability to enter information accurately and avoid issues of self-deception [3], as understanding quality in data input is one key aspect of information literacy in tracking.

In addition, tracking devices are not necessarily scientifically reliable. They remain unregulated, and there has been considerable speculation about their accuracy [40] and the extent to which valuable bodily data cannot be recorded with apps and devices [12]. So, an information literate individual would be aware of these issues and either find ways to take them into account or not use them at all. Yang et al [41] investigated how people themselves attempted to test trackers’ accuracy, though approaches to doing this were often flawed. Furthermore, the outputs of apps are not necessarily understandable by those who use them, since tracking demands the ability to interpret information outputs [19].

The extent to which people are aware of issues to do with the privacy of their personal data held in mobile apps or shared online is also an aspect of information literacy. Although market research in the United Kingdom has shown a majority of app users express concern about privacy and the extent to which apps share information about them, they are not always wary of using a social media account to access app functions, indicating a lack of awareness about potential reuse of data [6]. This parallels what has been dubbed the privacy paradox in social media, in that people are concerned about privacy but do risky things anyway [42]. This could be because they are not sure how to protect themselves, because they are not fully aware of the risk, or because of cynicism about having any privacy in a connected world. Further, research has found that many apps lack a data privacy statement, and often share data with third party organizations [43]. There have been several high-profile data breaches of consumer data, including in 2018, when 150 million users of the popular MyFitnessPal app were hacked [44]. A US study found that users were confident that apps kept their personal data secure [38], but other studies have found that users do have concerns about the privacy of their health data, particularly if data was sold to third parties [3,39]. A further area of concern is long-term access to data, whether because of the disappearance of platforms or the difficulty of exporting data when moving between devices. Thus, issues around data privacy and ownership constitute another area of information literacy relevant to tracking.

There are also aspects of information literacy bound up with appropriate data sharing. Research has shown that people are much more comfortable sharing activity and exercise data than they are sharing food and diet data [3,11,25]. Some studies have found positive perceptions of sharing exercise data, such as people enjoying a competitive relationship with friends and family relating to physical activity [11]. Digital health communities, where people share tracked data, have been identified as a motivating factor in increasing exercise [45]. Through these communities, it is possible for people to gain intimacy and social support through sharing, to benefit from crowd-sourced expertise, and to learn from others who have the same chronic condition [1,34]. However, other studies have shown that there are sensitivities with sharing tracked health data online, with some people considering sharing some health data (including diet information) to be completely unacceptable [3,25]. Unwanted automatic sharing of data with friends is a reason why people discontinue app use [38].

There seems to be a problematic relationship between people, their tracked health data and health professionals, as relatively few people report sharing data with a healthcare provider [36]. However, mobile apps that record diet have been identified as potentially useful, particularly for dietetic professionals [16], and people have seen value in being able to provide accurate data to healthcare professionals [10,38]. In particular, people with IBS are often advised to keep a diary of their symptoms and diet in order to share them with a doctor later [46,47]. However, healthcare providers often regard self-tracked data as unreliable, partly due to lack of diligence on the part of the patient and also due to their supposed unwillingness to admit to negative data [13]. There is also a perception among healthcare professionals that using apps in the context of managing a specific health problem could cause people to either undertake inappropriate or dangerous behaviors [19], or to promote obsessive or compulsive behaviors [13]. Patients feel that healthcare professionals are dismissive of their ability to collect accurate data or to know their own bodies [34].

In summary, the literature review identified that the adoption and use of tracking behaviors and technologies requires people to develop information literacy, both to understand the collection and interpretation of their own data, but also the social constraints around the sharing of that data. Understanding potential issues around privacy and security of data is also an aspect of information literacy in this context.

Thus, the research questions for this study were:

1. What do people in the three communities track and why?
2. What barriers to effective and safe use do they encounter, particularly in relation to information literacy?

This study is the first to investigate self-tracking for health and wellbeing in these specific communities and offers a novel comparative perspective on the attitudes and behaviors of people in regard to supporting their health. Framing tracking behaviors within an information literacy perspective focuses on users’ levels of competence in using information to meet their goals, which contributes to an increased understanding of the way people engage with information in the health arena.

## Methods

### Research Design

A questionnaire-based survey was used to gather insights about food and activity logging habits of three different populations of potential app users. Survey-based research designs have been previously used with success in other studies on food and activity logging [48-50]. The survey was composed of three main sections in which were eleven questions. Ten of these questions were closed-ended (three included a text box for additional comments) and one of them was a fully open-ended question to allow respondents to elaborate more on their experience as food and activity loggers. Closed-ended questions included demographic questions (Section A) such as age, gender, education level and an indication of the onset of their medical condition or experience as parkrunners, and questions related to the respondents’ views on logging (Section B) and information use (Section C) were included as both 5-point Likert scale statements and multiple-choice items. Prior to use in this study, the questionnaire had been pilot tested on a small sample of people representative of the three target populations, in order to guarantee consistency and improve readability. The study received ethical approval from the University of Sheffield Information School.

### Participants

The survey was distributed online via the websites parkrun, diabetes.co.uk and IBS Network in early 2018, and produced 143 valid responses from parkrunners, 140 valid responses from diabetes.co.uk, and 45 valid responses from the IBS Network. Each community received a tailored version of the survey, with questions such as the length of time respondents had been engaged in running, how long they had suffered from IBS, or when they had been diagnosed with type 2 diabetes tailored for each community. Response rates are not available as the survey was distributed by moderators of the communities in lieu of the authors. No incentives were offered to participants for completing the survey.

### Study Populations

The selection of these three specific communities is based on findings from a previous qualitative study [25], which highlighted how users with IBS and type 2 diabetes could benefit from food and activity tracking. In addition, that study identified a difference in tracking behaviors between diet and fitness tracking. Therefore, the present study aims to explore, in more detail, how very diverse groups of users make use of food and activity tracking functions.

Founded in the United Kingdom in 2004, parkrun is a not-for-profit organization that organizes weekly, timed, five kilometer runs in public spaces [51,52]. Events are free to enter and organized by volunteers, and parkrun’s ethos emphasizes inclusivity, as shown by how most participants were not regular runners before registering for parkrun. Evidence suggests that running has positive impacts on physical health and well-being, so mobilizing an inclusive community around running has significant potential public health benefits [53].

Type 2 diabetes is a lifelong condition which occurs when the human body cannot use insulin effectively and blood glucose (sugar) levels rise to higher than normal values [54]. Even though type 2 diabetes is mostly diagnosed in adults, it can also develop from a young age. Type 2 diabetes can be controlled if treated properly in its early stages by adopting a healthy lifestyle and healthier habits, such as exercising regularly, maintaining a normal weight and following a low carbohydrate diet [55]. If not managed correctly, though, it can lead to health complications such as heart disease, stroke, blindness, kidney failure and foot or leg amputations [56].

IBS has been defined as “a functional bowel disorder characterized by symptoms of abdominal pain or discomfort and associated with disturbed defecation.” [46] It is not understood as a single disease but instead as a range of physiological factors that contribute to commonly experienced symptoms [46]. While the causes are unknown, it is strongly linked to diet and stress, and diet changes are recommended as a way to control the symptoms that can vary enormously from person to person [47,57]. One commentator has estimated that around 11% of the global population has IBS [58]. Those self-identifying as suffering with IBS are predominately women [59].

### Data Analysis

All numerical data were entered into IBM SPSS Statistics version 24 and analyzed using descriptive statistics. The results of the 5-point Likert scale statements were aggregated to produce overall figures for agreement and disagreement responses. In addition, two-tailed independent samples *t* tests were performed to identify potential differences in attitudes between men and women, and also to differentiate between the levels of education of the respondents. The qualitative responses were manually coded independently by two members of the research team using thematic analysis [60], and the central themes surfaced for discussion alongside the quantitative data.

## Results

**Figure 1:**
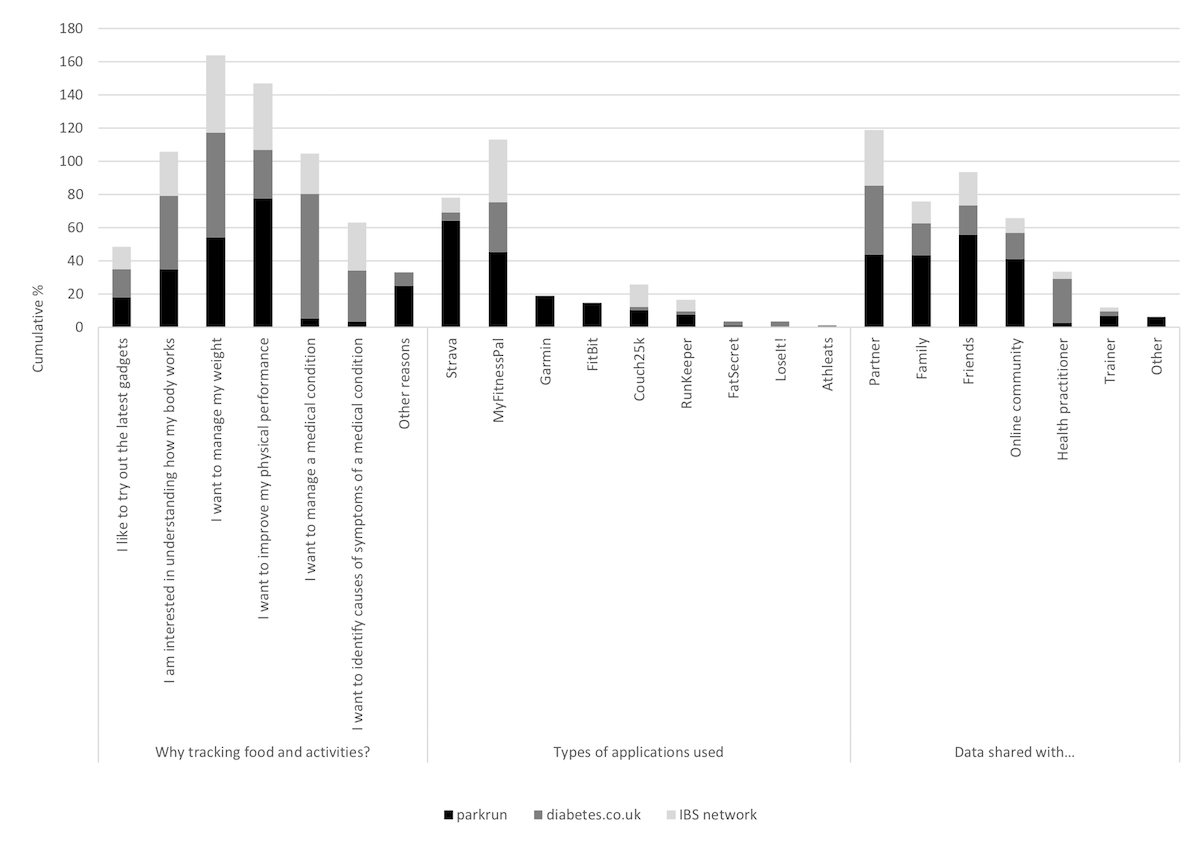
Apps used, reasons for tracking, and who data is shared with. IBS: irritable bowel syndrome.

**Figure 2:**
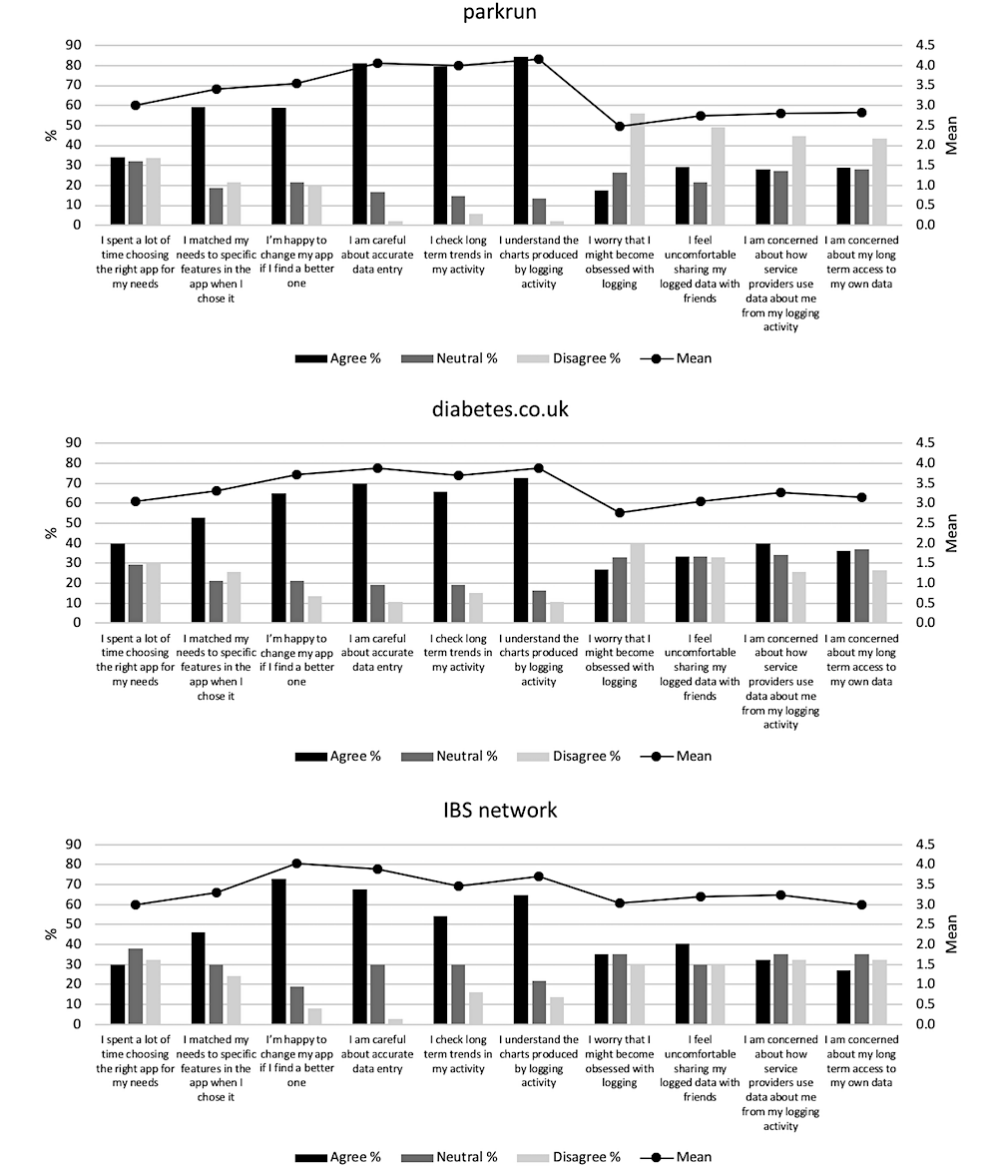
Views on app usage in the three communities. IBS: irritable bowel syndrome.

The demographic data obtained from the questionnaire (Section A) is reported in Table 1. A summary of the reported app usage (Section B) is reported in Figure 1. A summary of the responses to the Likert scale questions exploring opinions and behaviors relating to logging (Section C) are presented in Tables 2-4 and Figure 2. Tables 2-4 report on frequency of tracking behaviors in the three respondent groups, and Figure 2 presents opinions and views of respondents on their own tracking behaviors.

Selected quantitative and qualitative data are presented thematically below. Participants across the three communities tracked a variety of personal data related to exercise, food, and the body, and a variety of apps, automated devices and manual tracking procedures were used. To reflect this, the first three sections summarize the distinctive nature of tracking in the three groups, and this is followed by a thematic analysis of aspects of respondents’ information literacy that synthesizes data from across the groups.

**Table 1:** Demographic data from the respondents of the questionnaire.

Demographic characteristics		Parkrun, n (%)	Diabetes, n (%)	IBS^a^, n (%)
**How long have you been running for/have had type 2 diabetes/IBS?**				
	Less than 2 years	54 (37.8)	41 (29.3)	3 (6.7)
	2-5 years	51 (35.7)	48 (34.3)	12 (26.7)
	6-10 years	20 (14.0)	19 (13.6)	6 (13.3)
	More than 10 years	18 (12.6)	32 (22.9)	24 (53.3)
**Gender**				
	Male	45 (31.5)	57 (40.7)	4 (8.9)
	Female	97 (67.8)	83 (59.3)	41 (91.1)
	Other	1 (0.7)	0 (0.0)	0 (0.0)
**Age**				
	18-24 years	13 (9.1)	1 (0.7)	2 (4.4)
	25-34 years	19 (13.3)	2 (1.4)	14 (31.1)
	35-44 years	49 (34.3)	12 (8.6)	15 (33.3)
	45-54 years	44 (30.8)	43 (30.7)	7 (15.6)
	55-64 years	13 (9.1)	43 (30.7)	2 (4.4)
	65+ years	5 (3.5)	38 (27.1)	4 (8.9)
	Prefer not to say	0 (0.0)	0 (0.0)	1 (2.2)
**Highest level of Education**				
	Below GCSE^b^	0 (0.0)	6 (4.3)	0 (0.0)
	GCSE	11 (7.7)	24 (17.1)	4 (8.9)
	A level	24 (16.8)	14 (10.0)	11 (24.4)
	Undergraduate	62 (43.4)	44 (31.4)	15 (33.3)
	Postgraduate	43 (30.1)	43 (30.7)	13 (28.9)
	Prefer not to say	3 (2.1)	9 (6.4)	2 (4.4)

^a^IBS: irritable bowel syndrome.

^b^GCSE: General Certificate of Secondary Education.

**Table 2:** Frequency of tracking behaviors in parkrun respondents.

Behavior	Every day, n (%)	2-3 times per week, n (%)	Once a week, n (%)	Less than once a week, n (%)	In the past but not at the moment, n (%)	Never, n (%)
I use a food logging app	45 (31.5)	4(2.8)	1 (0.7)	1 (0.7)	36 (25.2)	56 (39.2)
I use a step counter	84 (58.7)	6 (4.2)	2 (1.4)	4 (2.8)	20 (14.0)	27 (18.9)
I use a device that records running	51 (35.7)	79 (55.2)	6 (4.2)	3 (2.1)	3 (2.1)	1 (0.7)
I track my heart rate and other vital signs	47 (32.9)	27 (18.9)	0 (0.0)	11 (7.7)	15 (10.5)	43 (30.1)
I keep a manual food diary	11 (7.7)	0 (0.0)	1 (0.7)	1 (0.7)	27 (18.9)	103 (72.0)
I track my weight	27 (18.9)	13 (9.1)	44 (30.8)	30 (21.0)	12 (8.4)	17 (11.9)
I track my mood	10 (7.0)	3 (2.1)	1 (0.7)	5 (3.5)	4 (2.8)	120 (83.9)

**Table 3:** Frequency in tracking behaviours in diabetes.co.uk respondents.

Behavior	Every day, n (%)	2-3 times per week, n (%)	Once a week, n (%)	Less than once a week, n (%)	In the past but not at the moment, n (%)	Never, n (%)
I use a food logging app	40 (28.6)	9 (6.4)	1 (0.7)	0 (0.0)	28 (20.0)	62 (44.3)
I use a step counter	58 (41.4)	6 (4.3)	2 (1.4)	2 (1.4)	23 (16.4)	49 (35.0)
I use a device that records running	22 (15.7)	7 (5.0)	2 (1.4)	1 (0.7)	9 (6.4)	99 (70.7)
I track my heart rate and other vital signs	27 (19.3)	14 (10.0)	7 (5.0)	17 (12.1)	10 (7.1)	65 (46.4)
I keep a manual food diary	24 (17.1)	6 (4.3)	3 (2.1)	3 (2.1)	32 (22.9)	72 (51.4)
I track my weight	40 (28.6)	26 (18.6)	33 (23.6)	20 (14.3)	12 (8.6)	9 (6.4)
I track my mood	19 (13.6)	10 (7.1)	6 (4.3)	6 (4.3)	16 (11.4)	83 (59.3)

**Table 4:** Frequency in tracking behaviours in IBS Network respondents.

Behavior	Every day, n (%)	2-3 times per week, n (%)	Once a week, n (%)	Less than once a week, n (%)	In the past but not at the moment, n (%)	Never, n (%)
I use a food logging app	8 (17.8)	1 (2.2)	0 (0.0)	1 (2.2)	16 (35.6)	19 (42.2)
I use a step counter	22 (48.9)	1 (2.2)	1 (2.2)	0 (0.0)	7 (15.6)	14 (31.1)
I use a device that records running	6 (13.3)	2 (4.4)	3 (6.7)	1 (2.2)	8 (17.8)	25 (55.6)
I track my heart rate and other vital signs	5 (11.1)	0 (0.0)	0 (0.0)	3 (6.7)	7 (15.6)	30 (66.7)
I keep a manual food diary	4 (8.9)	0 (0.0)	1 (2.2)	1 (2.2)	17 (37.8)	22 (48.9)
I track my weight	4 (8.9)	4 (8.9)	8 (17.8)	15 (33.3)	5 (11.1)	9 (20.0)
I track my mood	6 (13.3)	1 (2.2)	1 (2.2)	8 (17.8)	4 (8.9)	25 (55.6)

### Parkrunners

Parkrunners used more varied apps and devices to log than the other groups, reporting the use of least two apps on average, with some using as many as five. Unsurprisingly, parkrunners were the biggest users of devices that record running, with 35.7% (n=51) using one every day, and 55.2% (n=79) using one 2-3 times a week. Indeed, using or experimenting with one of these devices seems integral to the practice of running, as only 0.7% (n=1) of parkrunners had never used one. Recording of heart rate or other vital signs was also a popular aspect of tracking for parkrunners, with 32.9% (n=47) using one daily and 18.9% (n=27) using one 2-3 times a week. Parkrunners were primarily motivated by a desire to improve their performance (77.6%; n=111), and tracking was often used by parkrunners to compare their past performance to that of others:

I like to be able to track progress and have a goal because I tend to be results orientated.

Parkrun

Parkrunners reported tracking a variety of data related to their running practice, but this could be discontinuous and related to personal challenges:

I logged and referred to my steps daily as part of two challenges. One to do 10000 steps a day for one week for WI and another was to do 12,000 on average a day for the whole of Lent.

Parkrun

In addition, according to the Independent Samples *t* test results, among the parkrunner respondents, those with a higher level of formal education (undergraduate degree or above) reported the highest means of the whole sample in terms of checking long term trends of their activity (mean 4.09; SD 0.79) and understanding the charts produced by the logging activity (mean 4.25; SD 0.72).

### Diabetes

Tracking specific aspects of diet (eg, sugar intake) was frequent among diabetes respondents, with over half (55.7%; n=78) engaging in this tracking on a daily basis, and only a quarter (24.3%; n=34) having never tracked a specific aspect of their diet. Some respondents tracked heart rate or other vital signs either daily (19.3%; n=27) or 2-3 times a week (10%; n=14). Manual tracking was a feature of logging among the diabetes respondents (eg, in spreadsheets), with 75% (n=105) indicating that managing their condition was a motivation for their tracking:

I use my own log via an excel spreadsheet, that includes blood glucose testing results for each meal, food eaten and exercise on a daily basis. Helps me monitor my condition, track foods and/or exercise that helps or hinders control of my health.

Diabetes

My logging has been in physical journals and in computer documents. I use data, graphs etc. of my results when I am participating in a particular experiment concerning diet and activity, and my blood glucose levels, HBA1c, waist height ratio, hips, weight.

Diabetes

Diabetes respondents displayed a technical knowledge of their condition and the factors that they could log in order to manage it:

It is the main cause [that] my HCA1b is now in the 34 area which is normal non-diabetic level, arb intake around 280 grams a day.

Diabetes

Logging provided an element of control over the condition:

The process of logging helps me stay focussed.

Diabetes

Generally, I enjoy logging my daily actives and food intake it gives me a better understanding of how my blood sugar levels are impacted by diet, exercise and medication

Diabetes

### Irritable Bowel Syndrome

Although IBS is a condition that often involves sensitivity towards certain foods, surprisingly few IBS respondents were current users of food logging apps (17.8%; n=8). However, over a third of respondents (35.6%; n=16) had used one in the past, indicating that logging food could be valuable but possibly only over the short-term to identify triggering foods:

Great to start but cumbersome, especially if you have to log each ingredient every time. I tend to get bored and apps stop getting used.

IBS

IBS respondents were concerned about accuracy in data entry in common with the other groups, and the qualitative comments revealed a particular focus on perceived inaccuracies in food logging apps that could make the practice pointless:

I often feel that apps are lacking when it comes to logging food when you eat out or have a takeaway. I often find that many apps seem to suggest American based options so it can be difficult to find the right food. Sometimes it feels more like guess work than accurate tracking and logging.

IBS

Despite the interest in specialist diets (eg, FODMAP: fermentable oligo-, di-, monosaccharides and polyols) that have been shown to be effective in managing IBS symptoms [61], only 28.6% (n=40) of respondents (Table 4) used food logging for this purpose.

Although mood tracking was generally not a common aspect of tracking (see Tables 2 and 4), IBS respondents had the highest reported (35.5%; n=16) incidence of mood tracking from across the positive responses.

I tend to log my running activity so I can keep track of where I am with my progress. I also note in the tracking of how I felt on the day health / digestion wise so I can see if there is a link to anything in particular. I have had a good experience with tracking and will continue to do so in the future.

IBS

IBS respondents were motivated in their logging by a desire to manage weight (46.7%; n=21) and performance (40%; n=18). Surprisingly, managing a medical condition (24.2%; n=11) or identifying the cause of a symptom (28.9%; n=13) were not usually acknowledged as motives. The qualitative responses also underline the importance of weight management to logging practice for this group:

I started logging on and off in 2015. Logging my food intake has helped me to lose about 7kg and keep it off, taking me from borderline overweight to the middle of the healthy BMI range.

IBS

In contrast to the diabetes respondents, the logging practice for this group was not integral to their condition but more about maintaining general health through exercise and weight management.

### Information Literacy: Data Entry Quality

Overall, many participants demonstrated a strong awareness of issues around data quality, with 81.1% (n=116) of parkrunners, 70% (n=100) of diabetes and 67.5% (n=30) of IBS respondents agreeing with the statement “I am careful about accurate data entry”, thus recognizing the critical nature of data quality in their own inputs. The nature of food logging in particular requires people to be precise, including recording everything and weighing and measuring a complex range of ingredients in recipes. Interestingly, parkrunners (65.1%; n=93) and diabetes (52.9%; n=74) respondents were more likely than IBS respondents (68.2%; n=30) to be careful to log absolutely everything they ate if they used a food logging app.

The qualitative data revealed that people were well aware of the issues around accuracy of their own data input in the food logging context:

Difficult when local products are not in database and when item is scanned nothing is heard back. Recipes are tricky to enter.

Diabetes

Many apps are US based which means it's sometimes hard to find UK foods, but most of the time the barcode scanning works. Where it's less accurate is things like cherry tomatoes. I don't weigh them every time but I know an average weight that I use so I can go by quantity.

Parkrun

These complexities may explain why nearly all participants monitored their weight, yet the rate of food tracking was low. Only 17.8% (8) of IBS sufferers, 31.5% (45) of parkrunners and 28.6% (40) of diabetes respondents used a food logging app every day. Qualitative comments suggested why this was, as the practices of food logging and activity tracking had a very different feel. Food tracking was perceived to be worthy but time consuming, fiddly and potentially obsessive. Activity or running tracking was more automatic and seemed to be more inherently enjoyable, and often part of the enjoyment was data sharing. The nature of food logging meant it needed to be done multiple times in a day and would be checked frequently. Thus, food logging was more demanding, and as a consequence there were more complaints about the effort required:

Tedious but worthwhile. Any methods to make inputting information easier would be welcome.

Parkrun

A discourse of addiction or obsession was often used in relation to tracking, but it happened more often with food logging. It was perceived as more dangerous with food than activity, and thus, one person commented satirically on their obsession with recording running data:

I can be a bit obsessed with the “data” so much so that nothing happens until I have uploaded the info!!

Parkrun

The tone of the comment is lighthearted, but Independent Samples *t* tests conducted on gender among the parkrunner respondents show that females (mean 2.59; SD 1.06) are statistically significantly more worried about becoming obsessed with data logging than males (mean 2.22; SD 0.80). In addition, becoming obsessive about food emerged as a significant barrier to sustained use:

It's okay short term- long term tends to get obsessive and can, in my experience lead to disordered eating.

Parkrun

I try to balance keeping track of my numbers with not becoming obsessed by them.

Diabetes

The demanding requirement to gather accurate data throughout the day could be seen as creating this obsessive element. Thus, part of the information literacy of food tracking could be the management of risk around becoming obsessed with collecting data in a counterproductive way. Food tracking seemed often to be undertaken for short periods, probably for this reason. In contrast, comments on activity or running tracking often emphasized long term practice, enjoyment, how motivating it was, the online community element of it and how it was easy to do:

Run logging is fun and easy.

Parkrun

Where they did persevere with food logging, a number of solutions to data quality issues had been developed by participants, such as: (1) becoming a data creator (e.g. entering information from recipes into the app); (2) being particular about weighing food; and (3) modifying interpretations of the results to take into account any perceived inaccuracies.

I have created my own food entries in MyFitness Pal to be sure that my data is correct.

Diabetes

### Information Literacy: Interpretation of Tracking Information Outputs

The questionnaire results showed that around half (51.8%; n=72) of diabetes respondents had concerns about the extent to which apps took account of their personal metabolism, and a similar number of diabetes respondents (53.1%; n=74) and parkrunners (52.2%; n=75) had concerns about the quality of data entered by other users in the app. This reveals a critical awareness of the reliability of tracking apps in terms of information literacy. Concerns about data entry and the assumptions built into the app were a barrier to this form of tracking:

Haven't started using a food logging app as I find it mind boggling and difficult to use when it comes to home made food, plus their general approach to diet seems to fall onto the calorie deficit thinking whereas I view it more as quality of food i.e. not all calories are equal.

Parkrun

Regarding the interpretation of the information output of tracking, 121 (84.6%) parkrunners, 102 (72.8%) diabetes respondents and 29 (64.8%) IBS respondents said that they understood the charts produced by their apps. They also engaged closely with the data: 114 (79.7%) parkrunners said that they checked their long-term trends in activity, and so did 91 (65%) diabetes respondents and 24 (54%) IBS respondents. Again, qualitative responses suggested quite sophisticated use of apps, such as combining multiple devices or tracking different data in parallel:

I initially used My fitness pal to see how many calories were in specific foods and also to see how the calories balanced against manually inputted exercise. Then I got a Fitbit and linked the two. I am type 1 diabetic and am interested in keeping my weight at a healthy BMI. I also use Endomondo for logging runs and the training plan in it for my first half marathon in September.

Parkrun

Indeed, at least some participants had a critical sense of the limits of current designs of the tracking devices themselves:

Logging can be negative if a device wants you to move and you cannot, due to medical or personal reasons. Interfaces need to evolve and become more personal, flexible and compassionate.

Parkrun

You need to decide exactly what you want out of the process and not let an app designer dictate to you. Also don't get fixated on completeness and logging history. Keep asking the question: why is this useful?

Parkrun

### Information Literacy: Data Privacy and Ownership

Participants were asked about the extent to which they were concerned about how service providers used their logged data, with parkrunners most likely to be unconcerned (44.8%; n=64). Diabetes respondents were more worried about reuse of their data, with 24.3% (n=34) agreeing strongly and 40% (n=56) agreeing overall. The most common responses from IBS respondents were evenly distributed between agreeing, agreeing strongly or neutral, with 37.8% (n=17) choosing these options. Therefore, for each group, less than half of the respondents had concerns about potential reuse of their data.

Respondents were also relatively unconcerned about threats to long-term access of their data. Of the parkrunners, 43.4% (n=62) disagreed with the statement, “I am concerned about the long-term access to my data”, with 28% (n=40) answering neutral. Diabetes respondents were slightly more concerned, with around a third (36.4%; n=51) agreeing overall with the statement, but the most popular answer for this group was neutral (37.1%; n=52). IBS respondents were evenly split across agreeing or agreeing strongly (32.4%; n=15), neutral (35.1%; n=16) and disagreeing or disagreeing strongly (32.4%; n=14).

### Information Literacy: Information Sharing and Privacy

Different types of information seemed to be shared quite differently. Activity data was fairly freely shared, with the parkrunners as the greatest sharers of tracked data with friends and family, and by far the biggest sharers with online communities (41.3%; n=59). In the qualitative comments, data sharing was more commonly mentioned in relation to running activity, and seen as part of the enjoyment:

Seeing what my friends are doing (and knowing that they see what I do) is a major motivator for me in exercise and encourages me to get out and do things when I don't necessary feel like it. I also like statistics and tracking my performance.

Parkrun

Just a few qualitative comments revealed privacy concerns about running data:

I stopped using Strava because you could not hide runs from the public, which is a privacy concern as they could see or workout where I live and where I run on a regular basis.

Parkrun

IBS respondents shared the least data overall and were most likely to agree that data sharing made them feel uncomfortable, with 40.5% (n=18) agreeing or agreeing strongly and only 29.7% (n=13) disagreeing or disagreeing strongly. Specific to type 2 diabetes, women feel significantly more uncomfortable sharing data (mean 3.24; SD 1.27) than men (mean 2.75; SD 1.20). This probably reflects that, rather than activity data, they were collecting data about a medical condition or weight and diet, which was seen as more private. Sharing different types of data reflects an awareness of social norms surrounding tracked data.

A few strategies were mentioned as part of maintaining privacy, such as manual data tracking:

I strongly disagree with “cloud” based apps where I can't restrict data sharing. That's both for privacy, and also risk of losing access.

Diabetes

Similar sorts of sensitivities were reflected in who data was shared with. Partners were the most popular people to share data with across diabetes (41.4%; n=58) and IBS (33.3%; n=14) respondents, but friends were the most popular for parkrunners (55.9%; n=80). Diabetes respondents were the most likely to share data with a health practitioner, but the numbers were still quite low (26.4%; n=37). Less than 10% of the other two groups shared their data with an expert such as a trainer or doctor.

In summary, the results present the different varieties of tracking practice among the three communities studied and demonstrate that there are significant differences in motivations for tracking and uses for the data gathered. People who engage in self-tracking show evidence of information literacy through: ensuring data quality, understanding the information produced by tracking technologies and how this relates to their particular situation or medical condition, developing awareness of when and how to share their data, and developing an understanding of who has access to their data and the potential for sharing and reuse without their explicit consent.

## Discussion

### Overview

Tracking is used in different ways by different groups, but in all contexts, it is an information intense activity based on gathering, interpreting and managing data mediated by various devices and apps. The question of how information literate trackers are (how good their critical understanding of the information they are using is) thus becomes central to effective and safe tracking. This is one of the first papers to bring this perspective on tracking explicitly to the fore and complements research that has examined self-tracking from a Human-computer interaction perspective [12,62,63], a health behavior change perspective, [3] and a sociological perspective [13].

Respondents showed an understanding of the importance of their own accurate data entry, but also a skeptical awareness of its limits, especially in the context of food logging. In some cases, it was this critical understanding that led to nonuse, and in others, people found approaches to ensuring data quality or only used it intermittently. This is consistent with previous studies that show that simplifying diet and nutrition apps to make data entry less time consuming and more automatic was a key improvement desired by users [38]. It would make food logging much easier and also remove one aspect that created a fear of obsession, which is a common issue identified in self-tracking research [1,13].

While data accuracy is an important aspect of successful tracking, previous research into self-tracking has highlighted a tension between trusting data or trusting bodily sensations [12], with speculation regarding the relationship and potential value of each. In information literacy research, the role of corporeal information as a valuable source of information alongside social, epistemic, or formal sources is widely understood [22,25,64]. Conceptions of health information literacy indicate that assessing and evaluating information is a key activity, and that paying attention to the body and developing self-awareness supports the interpretation of other health information [26]. Diabetes respondents actively used information, often manually recorded, to manage their condition, which could be seen as an example of diagnostic tracking [35]. This extends conceptions of self-tracking beyond simply understanding a person’s relationship with technology to a broader understanding of their relationship with information [63]. Previous research into the information behavior of people with type 2 diabetes found that connecting information gathered from different objective and subjective bodily observations was an aspect of effectively managing the condition [34]. Integrating bodily information with app-related information has also been shown to be an important aspect of elite runners’ personal informatics practices [12]. Becoming information literate with regard to self-tracking, therefore, involves developing an understanding of how to integrate app data with corporeal information in order to achieve specific health goals.

Although the app MyFitnessPal was popular with participants in this study, perceived inaccuracies in either the app or one’s own measurements were also barriers to food logging. This is exacerbated by the acknowledged US bias of the food and measurements in the app’s database [16]. Many people, therefore, log food for only as long as it takes to either learn better food habits or to learn which foods trigger aspects of their condition. Discontinuing use can also occur due to the burdensome nature of tracking [38]. Information literacy therefore revolves around learning at what point the information needs have been met, and when to modify or discard the logging practice.

As regards interpreting information from tracking, respondents were confident in their own information literacy in interpreting the data output by the apps, and often used multiple devices in rather sophisticated tracking practices. They also made some critical comments on the questionable assumptions or expectations built into app design. This is consistent with previous research that has also found that users can be very capable of taking critical stance towards apps [65]. An area of rather more concern, consistent with some previous studies [40], is that many respondents were not worried about the use of their data by the platform or about long-term access to it, particularly in the current climate where app data is often widely shared with third parties without the express consent of the user [1,43]. Fortunately, some studies have found that data reuse is a serious issue for participants [3], and opinions may start shifting because of recent cases in the news.

Data sharing with friends and even in online communities was found to be central to activity tracking for many participants, which is consistent with previous research that has found sharing to be a fun aspect of tracking [12]. However, participants in this study were more reluctant to share data about health conditions, diet and weight, a point also identified by previous authors [3]. Information literacy research has found that sharing information about a chronic disease is a way for people to draw friends and family into their landscape and create a narrative about a disease [24], but this does not seem to be the case for participants in this study. Consistent with previous research [36,38], despite the potential benefits data was not often being shared with a trainer or doctor, which might reflect lack of interest by practitioners rather than trackers’ willingness to share data since they were already sharing with others, such as partners.

In summary, from an information literacy perspective, users seemed to be very literate in many aspects of logging practice. The relatively low use of food apps seems to reflect a critical perspective on the effort required to use them, their accuracy and their potentially obsessive effects.

### Limitations

This is a small-scale exploratory study, which only begins to identify the information literacy aspects of tracking behaviors for the three participant groups. All three groups of respondents reported a high level of prior education, which may not be representative of the populations as a whole. This may reflect a higher use of logging by higher socioeconomic groups [66], as more educated users seemed to have more confidence in their information literacy capabilities. In several respects we do not know how well the respondents represent the wider target population, partly because we do not have data about the demographics of the wider population and partly because respondents were self-selecting. The skew towards women among parkrunners, for example, may reflect greater willingness to participate in surveys rather than the actual proportions in the population [53]. The response rate from IBS sufferers was lower than for the other groups and so the results for this group should be treated with additional caution.

By definition, respondents to the questionnaire were current or past users of tracking apps. Many had not used or lapsed from use of particular forms of tracking, but the sample did not include those who had never tracked at all. This places a further limitation on the data as a means of understanding barriers to tracking in all contexts, however, studies of nonusers are inherently difficult.

The survey was based on asking participants to self-evaluate some of their information literacy skills (eg, their ability to understand charts produced by apps), so this may differ from actual competence. Overconfidence in information literacy is a known phenomenon [67]; however, levels of information literacy were implicit in many of the qualitative comments, which reflected complex, personalized practices of use.

### Conclusions and Implications

An information literacy perspective is of value because tracking is an information intensive activity, involving the user in entering data, in interpreting the information outputs of the device, and then managing access to said data. Effective and safe use of tracking thus depends on information literacy. This study showed that in three very different domains devices were used quite differently and levels of information literacy were also variable. In terms of understanding data entry quality, interpreting information, and appropriate sharing, respondents seemed to demonstrate good information literacy; however, a greater area of concern is around people’s lack of awareness of risks around platform use of data and continuity of access. This implies the need for much better public awareness around data ownership, and simplified privacy statements might assist in this. Organizations such as parkrun, Diabetes UK and the IBS network should consider this issue for their communities when they are providing advice and support about using mobile apps. The European Union’s General Data Protection Regulation is a move in a favorable direction in increasing protection of trackers’ privacy, but simple tools to extract data and maintain access to personal tracking data in the long-term are also needed. Additionally, there seems to be a gap in the market regarding mobile apps to support both the management of type 2 diabetes and IBS, given the reported manual tracking of one community and the pattern of app use and nonuse of the other.

## Abbreviations

eHealth: electronic health
FODMAP: fermentable oligo-, di-, monosaccharides and polyols
IBS: irritable bowel syndrome
